# Intra- and Inter-Limb Strength Asymmetry in Soccer: A Comparison of Professional and Under-18 Players

**DOI:** 10.3390/sports9090129

**Published:** 2021-09-13

**Authors:** Chris Bishop, Giuseppe Coratella, Marco Beato

**Affiliations:** 1 Faculty of Science and Technology, London Sports Institute, Middlesex University, London NW4 1RL, UK; c.bishop@mdx.ac.uk; 2 Department of Biomedical Sciences for Health, Università Degli Studi di Milano, 20126 Milan, Italy; giuseppe.coratella@unimi.it; 3 School of Health and Sports Science, University of Suffolk, Ipswich IP4 1QJ, UK

**Keywords:** football, strength, isokinetic, sports, prevention

## Abstract

(1) Background: the present study examined the isokinetic peak torque exerted by both knee extensors and flexors, anterior–posterior imbalance and the magnitude and direction of inter-limb asymmetry in professional and academy soccer players. (2) Methods: one hundred soccer players (professional = 50, elite academy = 50) volunteered to take part in this investigation. An isokinetic dynamometer was used to measure the knee extensor (quadriceps) and flexors muscle (hamstrings) torques of the limbs as well as inter-limb asymmetries—using a standard percentage difference equation. (3) Results: professional players exhibited significantly greater (effect size [ES] = *large*) strength levels in the quadriceps and hamstrings under both testing conditions, significantly higher (*small* to *moderate*) intra-limb ratio values for 60°·s^−1^ but not for the 300°·s^−1^ test condition, significantly (*small* to *moderate*) lower inter-limb asymmetry values for all test conditions, with the exception of the hamstrings at 60°·s^−1^ and the direction of asymmetry was *poor* to *slight*, indicating that limb dominance was rarely the same between groups. (4) Conclusions: this study shows that isokinetic assessments, i.e., peak torque exerted by both knee extensors and flexors and intra-limb ratio, and the subsequent inter-limb asymmetry, i.e., magnitude and direction, can differentiate between professional and academy soccer players.

## 1. Introduction 

Soccer players are required to perform a combination of movements where lower-limb strength and power are exerted maximally, such as jumps, sprints and changes in direction [1,2,3,4]. Despite the varied motor skills required in soccer, strength is a critical physical quality that underpins successful performance in these tasks [5,6,7,8]. Whilst numerous assessment methods exist, assessing the strength of the knee extensors and flexors may reflect, in part, the soccer players’ fitness status and may be used to discriminate elite from sub-elite players [9]. Additionally, the same study also observed that academy players had lower knee extensor and flexor strength than professional players [9]. In turn, these kinds of data may have implications for both on-field performance and injury risk [1,10]. Additionally, monitoring lower-limb strength over time could be helpful to develop the physical capacities of academy players by providing markers for the transition towards the professional level [3,9,11].

To accurately assess knee extensor and flexor strength, the isokinetic device is considered as the gold standard, since it allows the measurement of torque during both the concentric and the eccentric phases [12]. Despite its ability to only assess single-joint movements, this can be done at both high and low angular velocities [2,10,13,14]. Nonetheless, the torque assessed by knee extensors and flexors has been proposed as a potential tool to monitor the occurrence of non-contact injuries, such as muscle strains or non-contact anterior cruciate ligament (ACL) rupture [15,16]. Moreover, the ratio between the torque developed by the knee extensors and flexors has been taught as a means to assess lower-limb anterior–posterior balance (i.e., intra-limb asymmetry) [10,17,18]. Although its predictive power has recently been questioned, reduced eccentric knee flexor strength has been found as a potential marker for hamstring strain injuries [19]. Notwithstanding, the rationale for monitoring the anterior–posterior balance remains during a soccer player’s development [9], although it must be acknowledged that the risk of injury is multi-factorial in nature [20,21]

In addition to the lower-limb anterior–posterior balance, inter-limb asymmetries in strength should also be monitored for both performance and injury prevention purposes [1,16,22,23,24]. From a performance perspective, inter-limb asymmetry in both knee extensor and knee flexor strength has been reported to be associated with reduced linear (*r* = 0.34–0.47) and change in direction speed (*r* = 0.39–0.61) ability, while it does not seem to have any detrimental associations on jumping performance [1]. From an injury viewpoint, Kyritsis et al. (2016) reported that professional soccer players were four times more likely to re-rupture their ACL if they did not meet specific clinical discharge criteria [25]; one of these being quadriceps asymmetry being <10% when assessed at 60° s^−1^. Thus, and given that the aforementioned studies were both conducted in elite soccer players, it seems that between-limb imbalances in strength should be monitored from both a performance and injury risk perspective in this sport. On these bases, the present study examined the isokinetic peak torque exerted by both knee extensors and flexors in professional and academy soccer players. Additionally, both the anterior–posterior and the magnitude and direction of inter-limb asymmetry in lower-limb muscle strength were calculated. The hypothesis of this study is that professional soccer players should have a greater isokinetic peak torque exerted by both knee extensors and flexors than academy soccer players. Secondly, professional soccer players should present lower asymmetry values for all test conditions compared to academy players. However, a priory hypothesis was not done for the direction of asymmetry between groups. 

## 2. Materials and Methods

### 2.1. Participants

One hundred soccer players (professional = 50 [age = 23 ± 6 years, body mass = 77 ± 8.3 kg], elite academy = 50 [age = 18 ± 2 years, body mass = 73 ± 6.8 kg]) were enrolled in this investigation. Professional soccer players usually perform an average of 4 training sessions and 1 competitive match per week, with elite academy players usually performing a similar training and game frequency. However, the overall training and match volume have not been reported in this paper, therefore it is possible that the two groups were exposed to different volumes of training and match frequency. Goalkeepers were excluded from this study because of their different training and physical characteristics. All players that reported any injury at the moment of the testing protocol were excluded from the data recording [26]; this decision was taken by the club’s sport science department. Data were recorded by the club’s sport science department during their normal testing routine during the pre-season and were later on analysed for research purposes. The data analysis was conducted according to the Declaration of Helsinki for studies involving human participants. The procedures were previously approved by the university of Suffolk (UK) committee and were in line with the ethical standards in sports and exercise science. Players and guardians for the U-18 players provided written informed consent. 

### 2.2. Design

A cross-sectional study design was used in this study. A power calculation was performed using G-Power statistical software (Stuttgart, Germany). The study required the comparison of two independent groups, two tails test, and a *moderate* effect size was estimated because of previous research on the topic [9]. The sample size was estimated considering the probability to incur type 1 and type 2 errors using an α of 5% (error probability) and a ß of 80% (power), respectively. The needed sample size was 68 participants, which corresponds to a ß = 0.81. Therefore, the sample size used in this study had enough power to detect differences between the groups. Familiarisation sessions were not performed by players because the isokinetic test used in this study was part of the normal testing routine of the club and, therefore, players had already familiarised themselves with the instrument. Players avoided any vigorous training for the two days preceding the testing session and were instructed to avoid consuming any known stimulant (e.g., caffeine) or depressant (e.g., alcohol) substances for 24h before testing and, as well, were instructed to rehydrate *ad libitum* [27].

### 2.3. Isokinetic Measurements

An isokinetic dynamometer (Cybex Norm, Ronconcoma, NY, USA) was used to measure the knee extensor and flexor muscles torque of the limbs. The device was calibrated according to the manufacturer’s procedures. The testing procedure used was previously reported in the literature [9,10]. This test reported an excellent level of test–retest reliability (intra-class correlation coefficient [ICC] = 0.93–0.95) and a good level of technical error of measurement (around 4–5%) [14]. Players were seated on the dynamometer chair, with their trunks slightly reclined backwards and a hip angle of 95°. Two seatbelts secured the trunk, and one strap secured the tested limb, while the untested limb was secured by an additional lever. A standardized warm-up including 10 min of cycling at constant power (1 W per kg of body mass) on an ergometer (Sport Excalibur lode, Groningen, Netherlands) and separate 6 sub-maximal concentric repetitions for both quadriceps and hamstrings were performed. After that, peak torque was investigated at 60°·s^−1^ and at 300°·s^−1^ in the concentric modality [1]. The eccentric contraction was not assessed because players were not familiarised with such a contraction. Each testing modality consisted of 3 maximal repetitions and was separated by 2 min of passive recovery. Club sport scientists provided strong verbal encouragement to maximally perform during each trial. Both dominant and non-dominant limbs were tested in randomized order, with the dominant limb defined as the one preferred to kick a ball. The conventional Hconc:Qconc was then calculated and inserted into the data analysis [10].

### 2.4. Asymmetry Analysis

Mean inter-limb asymmetries were computed from isokinetic test using a standard percentage difference equation for both tests: 100/(max value) × (min value)^−1^ + 100, which has been suggested to be accurate for the quantification of asymmetries from unilateral tests [28]. In order to determine the direction of asymmetry (which provided an indication of limb dominance), an ‘IF function’ was added on to the end of the formula in Microsoft Excel: *IF(left < right, 1, −1), which ensured that the magnitude of asymmetry was not altered when different limbs performed in a superior manner [29,30].

### 2.5. Statistical Analysis

The Shapiro–Wilk test was used to verify the normality assumption (*p* > 0.05). Data were presented as mean ± standard deviation (SD). Independent *t*-tests were used to evaluate between-group differences in hamstrings and quadriceps peak torque, conventional Hconc:Qconc ratio was at 60°·s^−1^ and 300°·s^−1^. Significance was set at *p* < 0.05. Hedges *g* effect sizes with 95% confidence intervals were calculated to show practical differences between groups and were interpreted as: trivial: <0.20, *small*: 0.20–0.50, *moderate*: 0.50–0.80 or *large:* >0.80 [31]. Statistical analyses were performed using SPSS software version 24 for Windows 7, Chicago, IL, USA. Finally, Kappa coefficients were calculated to determine the levels of agreement for how consistently an asymmetry favoured the same side (direction of asymmetry) when comparing the different time points measured. This method was chosen because the Kappa coefficient describes the proportion of agreement between two methods after any agreement by chance has been removed [32]. Kappa values were interpreted in line with suggestions from [33], where: ≤0 = *poor*, 0.01–0.20 = *slight*, 0.21–0.40 = *fair*, 0.41–0.60 = *moderate*, 0.61–0.80 = *substantial*, 0.81–0.99 = *almost perfect* and 1 = *perfect*. 

## 3. Results 

Table 1 shows mean and SD data for all strength measures. Professional players showed significantly greater strength levels on all tests (on both limbs), as represented by *large* ES values (*g* range = 1.10–1.55). Intra-limb ratios showed *small* to *moderate* significant differences between groups for the 60°·s^−1^ condition (*g* range = 0.46–0.72), but not for the 300°·s^−1^ condition (*g* range = −0.09–0.33).

**Figure 1:**
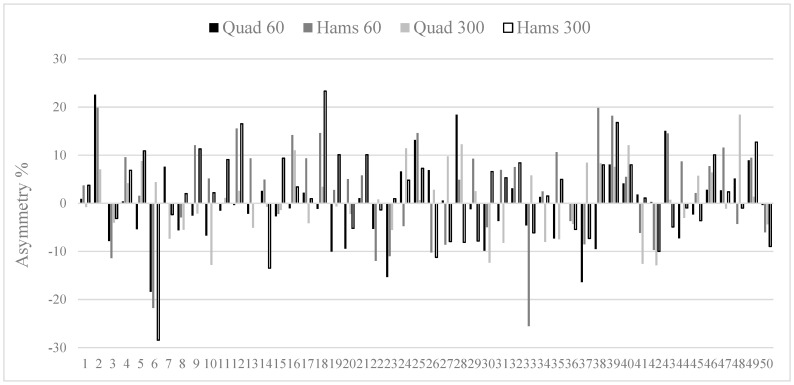
Individual inter-limb asymmetry data for professional players (*n* = 50). *Note:* above 0 indicates asymmetry favours the dominant limb and below 0 indicates asymmetry favours the non-dominant limb.

**Figure 2:**
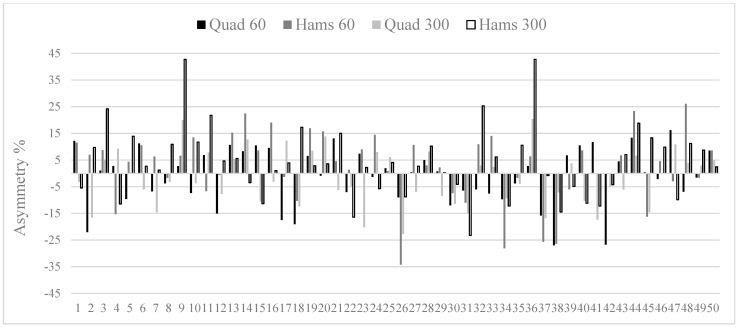
Individual inter-limb asymmetry data for under-18 players (*n* = 50). *Note:* above 0 indicates asymmetry favours the dominant limb and below 0 indicates asymmetry favours the non-dominant limb.

Table 2 shows mean inter-limb asymmetry data for all strength measures, with professional players exhibiting lower imbalances than under-18 players for all conditions. These *small* to *moderate* differences were all statistically significant for all strength measures (*g* range = −0.45 to −0.58), apart from hamstrings at 60°·s^−1^ (*g* = −0.23). Kappa coefficients were also computed between groups and showed *poor* to *slight* levels of agreement (K range = −0.16 to 0.04), indicating that it was rare for the direction of asymmetry to be similar between groups. Owing to the variable nature of inter-limb asymmetry, individual data have been provided for each player in the professional (Figure 1) and under-18 (Figure 2) groups. 

## 4. Discussion 

The aims of the present study were to examine the isokinetic peak torque exerted by both knee extensors and flexors in professional and academy soccer players and to calculate the magnitude and direction of inter-limb asymmetry for each group. The main findings were: (1) professional players exhibited significantly greater strength levels for the quadriceps and hamstrings under both testing conditions, (2) professional players showed significantly higher intra-limb ratio values for 60°·s^−1^ but not for the 300°·s^−1^ test condition, (3) professional players showed significantly lower inter-limb asymmetry values for all test conditions, with the exception of the hamstrings at 60°·s^−1^, and 4) the direction of asymmetry was *poor* to *slight*, indicating that limb dominance was rarely the same between groups. 

This study shows that professionals are significantly stronger in both the quadriceps and hamstrings, at slow (60°·s^−1^) and high (300°·s^−1^) velocity contractions (Table 1). While these results should not be a surprise [9], these data clearly show that when professionals have additional training and competing time, or the possibility to use more advanced strength training protocols, it results in *large* increases in strength compared to under-18 academy players. Another explanation of such differences between groups is due to the effect of age on these parameters, because younger players are likely to have not yet finished their overall muscle and skeletal development [11,34]. The relevance of this is especially important for the tested muscle groups in the present study, seeing as the hamstrings are the most commonly injured in soccer [35,36] and quadricep strength is a key requirement when returning from ACL injuries, which is another common injury in the sport [21,37,38]. Therefore, the assessment of the strength of the lower limbs using an isokinetic dynamometer can be useful to both physiotherapists and strength and conditioning coaches to monitor a player’s rehabilitation journey after knee injuries.

This study also provides data on anterior–posterior strength imbalances (i.e., intra-limb asymmetry) (Table 1). Interestingly, professional players showed significantly greater ratio values for the dominant and non-dominant limbs at 60°·s^−1^ (*g* range = 0.46–0.72), but not for the 300°·s^−1^ condition (*g* range = −0.09–0.33). Given the key difference in contraction speeds, these data highlight that academy players have a larger deficit in high-force compared to high-velocity contractions. Thus, from a practical applications perspective, it seems prudent to suggest that strength training in particular should be a focus for these academy players [4,21,39]. The authors believe that the knowledge of players’ anterior–posterior strength imbalances may help practitioners to individualise lower-limbs strength training programmes in order to reduce these side-to-side differences. Furthermore, given the emphasis that recent publications have placed on strength training for soccer players [21,40,41], this seems like an important suggestion. 

This study provides mean inter-limb asymmetry data for each muscle group across both contraction speeds (Table 2). Significant *small* to *moderate* differences were found between groups (*g* range = −0.45 to −0.58), with larger inter-limb differences evident in the academy players for all conditions except the hamstrings at 60°·s^−1^ (*g* = −0.23). This actually represents a somewhat unexpected finding, given that previous research has shown that meaningful differences in asymmetry are quite rare when assessing between groups or over repeated time points [23,29,34,42]. This is often due to the large within-group variability of asymmetry data, and our results support this notion, with the SD values being very high relative to the mean. However, in this instance, the difference in asymmetry is significant and when these data are viewed in conjunction with Table 1, the key take-home message is that academy players are weaker than professional players and that they have greater imbalances in both muscle groups, which could be considered a possible risk factor for future injury (Kyritsis et al. 2016). However, this study has analysed players from the same club and future studies should replicate the analysis performed with different soccer players to verify our findings. 

An additional focus of our investigation was to determine levels of agreement for the direction of asymmetry. Our results show that Kappa coefficients ranged from *poor* to *slight*, indicating that it was rare for any consistency in limb dominance characteristics to be evident between groups. Simply put, if professional players exhibited an asymmetry favouring the right limb (i.e., the right limb scored higher than the left), it was rare for academy players to show similar patterns. This provides further support for the task and population-specific nature of asymmetry, which has been suggested on numerous occasions in previous research [9,22,23,28,43]. In addition, when this is coupled with the large within-group variability of asymmetry (i.e., high SD relative to the mean), we have provided individual asymmetry data for professional (Figure 1) and academy (Figure 2) players. When viewing these figures, two key points stand out. Firstly, there appears to be little consistency in both the magnitude and direction of asymmetry for players (as evidenced by different sized bars which also go in both directions for many players). Secondly, the scale of the magnitude is much larger for academy players (+ and –45%). The relevance of this is that it does not seem uncommon for players to have a strength imbalance > 25%, which could be considered a potential risk factor for injury, based on previous suggestions [25], although future studies are needed to confirm our findings. 

This study is not without limitations. Firstly, this study used a sample of professional and academy soccer players from the same soccer club. This means that these findings cannot be applied to all soccer players and further research is needed to verify these results in other samples. Secondly, this study used only male soccer players; therefore, future studies should aim to examine a sample of professional and academy female soccer players and verify our results. Thirdly, this study uses a cross-sectional study, which analysed data from a soccer population at a specific point in time. Future studies should investigate seasonal variation of the parameters tested in this study, as well as how the training process can influence the variation of these parameters (e.g., asymmetries). Lastly, isokinetic assessments seem to be a reliable (concentric and eccentric peak torques, ICC = 0.93–0.95) [14] and useful testing method to verify lower limbs strength parameters and asymmetries in soccer players; however, it should be acknowledged that isokinetic dynamometers are very expensive and somewhat time-consuming to use. Consequently, many soccer clubs do not regularly use them; future studies may verify if other testing protocols and cheaper technologies could be more suitable for the assessments of lower limb strength and inter-limb and intra-limb asymmetries, e.g., flywheel devices [14,43,44]. 

## 5. Conclusions

This study found that professional players exhibited significantly greater strength levels for the quadriceps and hamstrings under both testing conditions, however, they only showed significantly greater intra-limb ratio values at low angular speed (i.e., 60°·s^−1^) and not at high speed (300°·s^−1^). Moreover, professional players showed lower inter-limb asymmetry values for all test conditions (except the hamstrings at 60°·s^−1^). In conclusion, this study shows that isokinetic assessments, i.e., peak torque exerted by both knee extensors and flexors and intra-limb ratio, and the subsequent inter-limb asymmetry, can differentiate between professional and academy soccer players. Strength and conditioning coaches should consider the use of isokinetic assessments in their practice and the subsequent analysis on asymmetries here proposed. This could give further insight into strength levels between academy players and professional players, as well as the existence of lower limb imbalances within their athletes. Finally, isokinetic parameters can be useful as a reference for planning strength and conditioning interventions during the season by referring back to benchmark scores and judging the efficacy of an intervention or block of training.

## Figures and Tables

**Table 1:** Mean ± standard deviation strength data for both limbs, and Hedges *g* effect sizes with 95% confidence intervals (CI).

Strength Measure	Professionals (*n* = 50)	Under-18 (*n* = 50)	Hedges *g* (95% CI)	*g Descriptor*
*Quadriceps 60°·s^−1^:*				
Dominant	297.9 ± 45.4	241.8 ± 39.4	**1.32 (0.95, 1.67)**	*Large*
Non-Dominant	299.3 ± 46.2	245.4 ± 39.6	**1.26 (0.89, 1.61)**	*Large*
*Hamstrings 60°·s^−1^:*				
Dominant	184.0 ± 39.0	135.9 ± 30.3	**1.37 (1.01, 1.73)**	*Large*
Non-Dominant	178.4 ± 34.9	131.5 ± 24.2	**1.55 (1.18, 1.93)**	*Large*
*Intra-limb Ratio 60°·s^−1^:*				
Dominant	0.62 ± 0.09	0.57 ± 0.12	**0.46 (0.13, 0.79)**	*Small*
Non-Dominant	0.60 ± 0.07	0.54 ± 0.10	**0.72 (0.38, 1.05)**	*Moderate*
*Quadriceps 300°·s^−1^:*				
Dominant	152.2 ± 19.6	124.3 ± 19.9	**1.42 (1.04, 1.77)**	*Large*
Non-Dominant	151.2 ± 19.7	126.5 ± 18.9	**1.27 (0.90, 1.62)**	*Large*
*Hamstrings 300°·s^−1^:*				
Dominant	99.5 ± 17.7	82.0 ± 14.5	**1.10 (0.74, 1.44)**	*Large*
Non-Dominant	97.4 ± 14.5	78.0 ± 13.8	**1.37 (1.00, 1.73)**	*Large*
*Intra-limb Ratio 300°·s^−1^:*				
Dominant	0.66 ± 0.10	0.67 ± 0.11	−0.09 (−0.41, 0.22)	*Trivial*
Non-Dominant	0.65 ± 0.09	0.62 ± 0.09	0.33 (−0.01, 0.65)	*Small*

**N.B**: effects sizes in bold signify significant difference between groups (*p* < 0.05).

**Table 2:** Mean ± standard deviation inter-limb asymmetry data (%), Hedges *g* effect sizes with 95% confidence intervals (CI) and Kappa coefficients (assessing levels of agreement in the direction of asymmetry between groups).

Asymmetry Variable	Professionals (*n* = 50)	Under-18 (*n* = 50)	Hedges *g* (95% CI)	*g Descriptor*	Kappa
Quadriceps 60°·s^−1^	5.9 ± 5.5	8.6 ± 6.4	**−0.46** **(−0.79, −0.13)**	*Small*	0.04 *(slight)*
Hamstrings 60°·s^−1^	8.8 ± 5.7	10.4 ± 8.3	−0.23 (−0.55, 0.09)	*Small*	−0.16 *(poor)*
Quadriceps 300°·s^−1^	5.7 ± 4.4	8.9 ± 5.6	**−0.58** **(−0.92, −0.25)**	*Moderate*	−0.05 *(poor)*
Hamstrings 300°·s^−1^	7.1 ± 5.7	10.6 ± 9.2	**−0.45** **(−0.78, −0.12)**	*Small*	−0.13 *(poor)*

**N.B**: effects sizes in bold signify significant difference between groups (*p* < 0.05).

## Data Availability

The data presented in this study are available on reasonable request from the corresponding author.
